# Ear Cough

**Published:** 1893-09

**Authors:** A. G. Aldrich


### Abstracts.

### EAR COUGH.

Too often neglect of detailed examination leads to diagnostic errors and unsuccessful treatment, when symptoms are directly manifested; and far too often does this occur when disorders at remote points are manifested through the reflexes.

It is believed that the occasions are infrequent when the aurist should be consulted for the explanation of cough. This I believe to be true, but that there are more cases than generally observed, I also believe to be true. During the last thirteen years of general and special practice, I have in many cases found it quite impossible to examine the ear without exciting a cough, the pressure of the speculum alone many times causing quite a paroxysm of coughing. I have also observed many cases where inflammation of the auditory canal has kept up an irritable cough, which has subsided upon the proper treatment.

One very interesting case of ear cough, which I wish to report, in order to add to the literature upon this subject, is that of Mrs. L., a clergyman's wife, who consulted me in June, 1886, for deafness of the right ear. I observed, during the consultation, that she suffered from a harsh rasping cough. She informed me that she had been coughing for a year, and had consulted several physicians, each making a different diagnosis, and that at the present time she was under the care of a gynecologist who pronounced it of reflex uterine origin. Upon inspection, I found the auditory canal filled with what seemed to be inspissated cerumen. I succeeded in removing the mass, which I found to be a cockroach entirely incrusted with cerumen. Her deafness was, of course, relieved, the cough ceased immediately, and has never returned. I think it advisable, in all cases of intractable cough of apparent unknown origin, to which is applied that vague and unscientific appellation, "irritable cough," that the auditory canal should be examined. I have observed that most individuals suffering from ear cough have been neurasthenics, but, as in reflex affections of other organs, where the cause seems skeptically slight, so in this affection we must give due attention to the reflexes of aural origin. The exciting cause in all cases coming under my observation has been pressure causing irritation. Opportunities for studying aural reflexes have been very limited for specialists in otology, as the cases fall more frequently into the hands of the general practitioner. The subject is not, nor will it ever be, as voluminous as that of some other reflexes, but it is interesting and will add its mite to the general study of reflex phenomena.—*A. G. Aldrich, M. D., in Ophthalmic Record, August, 1893.*

